# Exploring effect of chitosan on antioxidant system and hypericin content in *Hypericum perforatum* L. under various irrigation regimes

**DOI:** 10.1186/s12870-025-06887-y

**Published:** 2025-07-02

**Authors:** Rayhaneh Amooaghaie, Nafiseh Rajaie

**Affiliations:** 1 Plant Science Department, Faculty of Science, Shahrekord University, Shahrekord, Iran; 2 Biotechnology Research Institute, Shahrekord University, Shahrekord, Iran

**Keywords:** Antioxidant enzymes, Chitosan, DPPH, Lipid peroxidation, St john’s wort, Phenolics

## Abstract

The beneficial effects of chitosan on stress tolerance and secondary metabolism have been documented in a few medicinal plants. However, the impact of chitosan on water deficit tolerance in St John’s Wort (*Hypericum perforatum* L) remains largely unknown. Therefore, a field experiment was executed to assess the effect of foliar spraying various chitosan concentrations (0, 100, 200, and 400 mg L^−1^) under irrigation intervals 7 (normal), 10 (mild water stress), and 13 (severe water stress) days. When irrigation intervals increased, biomass substantially decreased in both harvests. However, hypericin content increased at mild water stress, whereas it decreased under severe water stress. Foliar application of chitosan increased biomass, total phenol content )TPC( and hypericin content under all irrigation intervals. The highest hypericin content and TPC were verified by spraying 200 mg L^−1^ chitosan under the mild water stress that corresponded with the most 2,2-diphenylpicrylhydrazyl (DPPH) scavenging activity in the leaves. The foliar application of chitosan improved relative water content, chlorophyll *a*,* b* contents and decreased the content of H_2_O_2_ and malondialdehyde in plants grown under irrigation intervals of 10 and 13 days. The compensatory effect of chitosan was due to enhancing the activities of catalase and peroxidase and increasing proline content in leaves. However, 400 mg L^−1^ of chitosan negatively affected most of the aforementioned attributes in St John’s Wort. Collectively, the results indicate that foliar spraying with an appropriate concentration of chitosan may be a promising solution for improving the productivity and pharmaceutical value of medicinal plants under water deficit.

## Introduction

Due to climate change, drought stress is a major environmental challenge in the 21 st century and many countries will face more water shortage problems in the coming years. Insufficient irrigation is one of the most important limiting factors for plant productivity in arid and semi-arid countries with limited water resources [1, 2]. Water deficit decreases the osmotic potential, increases reactive oxygen species (ROS) generation, and causes oxidative damage in cells. These ROS lead to the peroxidation of membrane lipids, protein denaturation, and inactivation of enzymes and finally disturb physiological, and biochemical processes [1, 3]. The survival of plants under water stress plants depends on their ability to induce various defense mechanisms. The plants can protect themselves against oxidative stress by activating antioxidant enzymes such as ascorbate peroxidase (APX), catalase (CAT), peroxidase (POD), and superoxide dismutase (SOD), and producing non-enzymatic antioxidants such as phenols and flavonoids [1–5]. The biosynthesis of phenolic compounds through phenylpropanoid pathway is vitalized by various abiotic stresses and is considered a constitutive biochemical defense strategy [5–7]. Phenolics can contribute to mitigating oxidative stress due to their antioxidant potential and capacity for acting as electron and hydrogen donors [6–8]. It is known that exposure to mild drought stress may strengthen secondary metabolism in medicinal herbs and lead to some economic benefits for growers [5, 6, 9]. Several studies explored that water stress increased rutin, betulinic acid, and quercetin in *Hypericum brasiliense* [10], curcumin in *Curcuma longa* L [11]. and artemisinin in *Artemisia* [12]. However, defensive mechanisms might be insufficient, and the productivity and quality of medicinal herbs decrease under severe drought stress [5–7]. Therefore, finding innovative solutions for enhancing plant growth and secondary metabolite production under stress conditions is a critical area of research in modern agriculture and plant sciences. One of the most promising strategies to address this challenge is the use of natural biostimulants, which have demonstrated significant potential in improving stress tolerance and optimizing secondary metabolic pathways in plants [13]. In this context, the exogenous application of biodegradable and biocompatible compounds such as chitosan to enhance plants’ resilience to drought and increase secondary metabolites in medicinal plants has garnered significant attention in recent decades [14].

Chitosan is a natural and polycationic polymer produced by the alkaline N-deacetylation of chitin. This amino polysaccharide can promote plants’ growth and enhance the yield and secondary metabolism in crops and medicinal herbs [14, 15]. For instance, foliar spraying with chitosan increased chlorophyll fluorescence, photosynthesis, the accumulation of rosmarinic acid, anthocyanins, and the level of total phenolic compounds (TPC) in *Ocimum basilicum* L. and *Melissa officinalis* L [16]. This natural polymer exists in the cell walls of many pathogenic fungi and for this reason, plants respond to it by activating defensive genes and enzymes and eliciting secondary metabolism [15]. Therefore, chitosan can be used as a potent elicitor of secondary metabolism and an inducer of defensive responses in plants under abiotic and biotic stresses [17–21]. Previous studies revealed the potent ability of chitosan as a biostimulator to enhance the yield and quality of many crops and herbs under water stress [11, 12, 19, 22]. Li et al. [23] using transcriptomic and metabolomic analysis elucidated that chitosan upregulated stress signaling, carbohydrate and amino acid metabolism, tricarboxylic acid cycle, ascorbate-glutathione pathway, and flavonoid metabolism in *Trifolium repens* under drought stress. Foliar application of chitosan mitigated the negative effect of water stress on dry matter yield, and increased essential oil content and total phenol content in *Thymus daenensis* [24] and *Hyssopus officinalis* L [24]. Akhtar et al. [25] reported that chitosan application significantly increased the content of pigments, transpiration, and photosynthetic rates, water use efficiency, stomatal conductance, and the activities of antioxidant enzymes resulted in increased biomass and drought tolerance in pot marigold. Foliar spraying with chitosan changed essential oil composition and enhanced total phenol and flavonoid contents, and antioxidant activity in *Salvia officinalis* L. extract under various irrigation frequencies [26]. Safikhan et al. [27] also found that soil application of chitosan improved chlorophyll content, plant growth, and the biomass of *Silybum marianum* (L.) Gaertn. under salinity stress due to increasing proline content, and enhancing antioxidant enzyme activity in leaves. Khodadadi et al. [28] using transcriptomic and metabolic techniques found that both water stress and chitosan stimulated methylerythritol phosphate (MEP) and mevalonate (MVA) pathways and advanced terpenoid and polyphenolic components in essential oil of *Salvia yangii* and *S. abrotanoides.* It has been suggested that binding chitosan to specific cell membrane receptors can promote signaling cascades with the participation of signal molecules such as hydrogen peroxide (H_2_O_2_), nitric oxide, and phytohormones which in turn trigger stress-responsive genes and enzymes in cells [17, 20, 29, 30].

Although considerable advancements in exploring the impact of chitosan on the quality and productivity of medicinal herbs have been obtained, limited studies have documented the beneficial effects of chitosan on the secondary metabolism of Hypericum species in tissue culture [31, 32]. *Hypericum perforatum* L. (is known also as St John’s Wort) is among the most important species of this genus and its flowering aerial parts are widely utilized for therapeutic purposes [33]. This herb is extensively applied in traditional and modern medicine for its antidepressant properties, due to compounds such as hypericin, pseudohypericin, and hyperforin. Hypericin is a naphtodianthrone reputed as the main quality marker for this herb that is synthesized in the dark glands and distributed throughout the aerial parts, with the lowest levels in the vegetative stage and, peaking during flowering and seed-ripening stages [34]. Hypericin has gained a lot of attention in the pharmaceutical industry due to its high therapeutic properties, such as its effects against flocculation mild stress-induced depression, and metabolic dysfunction along with anti-tumor, antiviral, antibacterial, and antioxidant activity and its potential for photodynamic diagnostics [33]. The plant also contains antioxidant flavonoids (e.g., kaempferol, quercetin) and phenolic acids, which contribute to its antioxidant, antiviral, wound-healing, and hepatoprotective activities [32–34]. Due to the growing demand in the food and pharmaceutical industry for bioactive compounds derived from St John’s Wort, raw materials are primarily sourced from field-grown plants [33, 34]. St John’s Wort is a perennial plant herb whose biomass of flowering tops is limited in the first cultivation year, but reaches its maximum potential in the second year, and can remain high during the third year [34]. The contents of hypericin and phenolic compounds are influenced by physiological and environmental factors such as climate, temperature, genotype, developmental stage of the plant, and harvesting time [35, 36]. This underscores further research to find solutions for optimizing hypericin yield and improving the plant’s resilience under challenging field conditions. The current literature provides limited evidence regarding the impact of abiotic stresses on hypericin content. While *H. perforatum* is recognized as a well-adapted herb to a variety of challenging environments including arid and semi-arid conditions [37], prolonged water stress during pre-flowering stages has been shown to reduce dry mass yield and alter the phytochemical composition of this herb [38]. Several studies revealed that chitosan enhanced water stress tolerance and secondary metabolism in *Origanum majorana* [39], *Cichorium intybus* L [40], and *Dracocephalum kotschyi* [41]. A study also depicted that chitosan supplementation increased xanthone production and VOC biosynthesis in St John’s Wort (*Hypericum perforatum* L.) root cultures [42]. However, the effects of foliar spraying chitosan on field-grown *H. perforatum* plants under various water regimes remain unexplored. Given the beneficial effect of chitosan on stress tolerance and secondary metabolism in medicinal plants, it is hypothesized that foliar application of chitosan may improve water deficit tolerance and enhance the accumulation of hypericin and phenolics as key quality markers in field-grown *H. perforatum* plants.

## Materials and methods

### Plant cultivation and performance of the experiment

St John’s Wort (*Hypericum perforatum*) seeds were obtained from Pakan Bazr Company in Isfahan, Iran. In the first year, seeds were sown in pots filled with a cocopeat and perlite in February 2013 and pots were transferred to greenhouse at 16/8 h light/dark, 25/18°C thermoperiod and 75% relative Humidity. At first, pots were watered daily with tap water and after seedling establishment were irrigated two or three times per week based on the water requirements of plants to prevent wilting. In March 2013, seedlings were transplanted in an open field within the research farm of Gol Daru Company at Kelishad, Iran (x = 550043, Y = 3598801). Soil characteristics were comprised of pH 6.95; 0.2% O.C, 0.02% total N, 16.5 mg kg^−1^ available P, 205 mg kg^−1^ available K, and E.C. 1.5 dS/m. During soil preparation, 30 tons ha^−1^ of rotten animal manure, 200 kg ha^−1^ phosphorus and 200 kg ha^−1^ potassium were added and mixed with the soil. In addition, for weed control, 3 L ha^−1^ Trifluralin was applied before transplant. No intervention against pests was needed.

In the first year, all plots were watered every 7 days throughout spring and summer, from the transplant to full flowering time (i.e., harvest time) to fully provide the water requirements of plants. Then, aerial parts of plants were cut at ground level, in June, 2013 and roots remained for regrowth in next year. No treatment or test was done on these plants. It is noteworthy that the biomass yield of flowering tops in the first year of cultivation is relatively low and based on the natural growth pattern; St. John’s Wort plants achieve their mature size and morphology by the second year which aligns with typical increases in flowering tops and hypericin content in this herb [34]. Therefore, the treatments of the current study were implemented in the second year.

In the second year of plant growth (i.e. 2014), the regrowth process was done in two distinct seasons. The first season commenced in late February and finished on June 15th. The second season spanned from June 17th to September 30th. Therefore, two harvests were executed, on June 15th and September 30th. At first of both seasons, 200 kg ha^−1^ Urea and 20 kg ha^−1^ NPK fertilizer (20-20-20) were added to support the regrowth of plants. The climatic characteristics recorded in the location of experiment during two growing seasons are represented in Table 1.

**Table 1:** Monthly average temperature, precipitation, and relative humidity during February-September 2014

	February	March	April	May	June	July	August	September
Temperature	5.5	10.1	14	19.2	24.4	29.2	28.5	24.1
Precipitation	10	57.2	16.3	11.8	0.7	0.0	0.0	0.0
Humidity	59	46	39	41	28	21	24	25

A split-plot experiment was carried out with a randomized complete block design (RCBD) and three replications. Each experimental plot was 2.5 × 6.5 m, and plants were grown in 5 rows, with a spacing of 30 cm in rows 40 cm apart per replication. Adjacent subplots, main plots and replications (blocks) were 1.0, 2.0, and 2.5 m apart, respectively. Various irrigation intervals were performed in the main plots and foliar spraying with different concentrations (0, 200, and 400 mg L^−1^) of chitosan (purchased from Sigma–Aldrich Co., Steineheim, Germany) were done in the subplots. It is worth noting that these chitosan concentrations corresponded with other studies that indicated 100–500 mg L^−1^ chitosan was beneficial for enhancing stress tolerance and secondary metabolites in various plants [16, 18, 21]. After dissolving chitosan in acetic acid, various concentrations were achieved by diluting it with distilled water. Based on the report of Agricultural research center of Iran, normal irrigation for this plant is every 7 days. Therefore, various irrigation intervals in the main plots were included: every 7, 10, and 13 days which respectively corresponded with 83–86% in field capacity, 69–73% F.C. and 48–52% F.C. based on measuring soil moisture by a TDR device. Considering these levels of field capacity, RWC in plants and our observations from plant appearance, we described these intervals as normal irrigation, moderate water deficit, and sever water deficit for this plants.

**Fig. 1:**
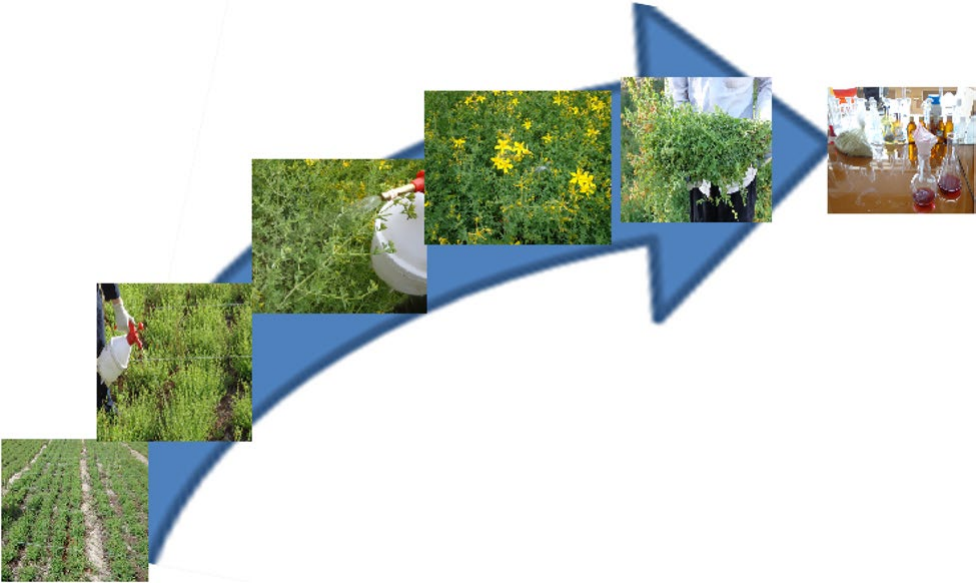
Images showing cultivation, chitosan spraying, harvest and hypericin extraction

In the first season of plant growth in 2014 (i.e. the second year of plant growth), plants restarted vegetation in late February. Water deficit was not performed in the first month of the growth cycle and all plots were irrigated at 7-day intervals. On 30 March, when plants reached an average of 30 cm in height, irrigation regimes and foliar spraying with chitosan simultaneously were begun. The foliar spraying chitosan was done three times, the first time on 30 March in the early vegetative stage, the second time on 15 May before the flowering stage, and the third time on 1 June when plants were in the 25% flowering stage. The chitosan solutions (60 mL/plant the first time, and 120 mL/plant in the second and the third times) were sprayed by a hand sprayer on the whole parts of the plants at dew point i.e. early morning (See Fig. 1). One day after the last spray of chitosan, fresh leaves were collected and immediately frozen in liquid nitrogen and kept at − 80 °C for various biochemical analyses. Then, all plants were cut at ground level, at full flowering stage on 15 June. Samples of five plants were randomly taken per treatment from the middle of plots and were open-air dried in the shade for one week at room temperature. To obtain the herbal yields, the average aerial parts dry weight of 5 plants was reported as biomass per plant.

In the second growing season of 2014, vegetation resumed after the initial cutting. To facilitate rapid regrowth, all plots were irrigated at 7-day intervals over a 2-week period. Then, plants in main plots again grew with the same irrigation regimes every 7, 10, and 13 days, and foliar spraying with chitosan was done three times at 30, 55, and 70 days after regrowth. Finally, plants were harvested in 30 September 2014 for the second time and after drying, biomass and hypericin content were measured in them.

### Evaluation of water status in leaves and proline content

The relative water content (RWC) in fresh leaves was determined by the Eq. 1:

1 $$\%\mathrm{RWC}=\left[\frac{\left(\mathrm{FW}-\mathrm{DW}\right)}{\left(\mathrm{TW}-\mathrm{DW}\right)}\right]\times100$$

Where DW is the dry weight (after drying in an oven at 75 °C), FW is fresh weight, and turgor weight is the weight of leaf samples after floating the leaves in water for 7 h [43].

The Bates et al. method [44] was adopted to quantify the proline contents in leaves. Briefly, after macerating leaf samples in 3% sulfosalicylic acid, the homogenate was centrifuged at 4 °C. Then, the mixture of supernatant, glacial acetic acid, and ninhydrin reagent was heated for 40 min. After cooling and adding toluene, the mixture was vortexed and incubated at room temperature. The optical absorbance was recorded at 520 nm, and the proline concentration was estimated using the standard curve.

### Measurement of chlorophyll

After extracting fresh leaf samples using acetone (80%, v/v), the optical density of the homogenate was recorded at 663 and 645 nm. Chlorophyll *a*,* b* (Chl *a*, Chl *b*) contents were quantified by Eqs. 2 and 3 suggested by Lichtenthaler and Wellburn [45].

2 $$Chl.\;a\;=\;\frac{\left[\left(12.21\;\mathrm A663\right)-\left(2.81\times\mathrm A646\right)\times\mathrm V\right]}{1000\;\mathrm W}$$

3 $$Chl.\;b\;=\frac{\left[\left(20.13\;\mathrm A646\right)-\left(5.03\times\mathrm A663\right)\times\mathrm V\right]}{1000\;\mathrm W}$$

### Estimation of hydrogen peroxide and malondialdehyde contents

Heath and Packer’s method [46] was adopted to measure malondialdehyde (MDA) content as an indicator of membrane damage. Leaf samples were macerated in trichloroacetic acid and centrifuged at 10,000 g for 5 min. The mixture of thiobarbituric acid, tricholoroacetic acid, and supernatant, was incubated and centrifuged. The MDA content was computed by reading the optical density at 600 and 532 nm and using an extinction coefficient of 155 mM^−1^ cm^−1^.

H_2_O_2_ content was quantified using a KI reagent according to the procedure adopted by Velikova et al. [47]. The H_2_O_2_ content was calculated by recording the optical absorbance at 390 nm and using a standard curve.

### Assaying the antioxidant enzyme activities

First, frozen leaves were homogenized using an extraction buffer. After centrifuging, Guaiacol peroxidase (POD) and catalase (CAT) activity were determined in the resulting supernatant by the method adopted by Heidari et al. [20].

CAT enzyme activity was evaluated based on the decline in optical absorbance at 240 nm per 1 min and the extinction coefficient of 39.4 mM^–1^ cm^−1^. POD activity was measured using absorbance variations of the reaction mixture at 470 nm for 1 min and an extinction coefficient of 26.6 mM^−1^ cm^−1^.

### Quantification of hypericin content

First, flowering tops were dried in the shade and then, powdered and soaked in acidic acetone for 2 h. The resulting solution was filtrated twice and then dried by a vacuum dryer. The residue was dissolved and diluted to 100 ml with methanol. The absorbance of the solution was read at 587 nm compared to methanol as blank. Hypericin content was calculated by the following equation [48]:

4 $$Hipg\%\;=\;\frac A{780}.\frac{100}{780\;m}$$

Where: A is the optical absorbance of the extract, m is the weight (g) of the sample applied for extraction, and 780 is the specific absorbance of hypericin at 587 nm.

### Quantification of total phenolic content and DPPH scavenging activity

To measure phenolic content, 1 g of powdered and dried leaves was soaked in methanol 80% and 10 min centrifuged (10000 ×g) at 4 °C. After 15 min incubating the mixture of deionized water, supernatant, Folin-Ciocaltu reagent and sodium carbonate 2%, the absorbance of the solution was read at 750 nm. Total phenol content (TPC) was expressed as mg gallic acid equivalent per gram dry weight [43].

The scavenging capacity of DPPH radicals was assayed using the method adopted by Amooaghaie et al. [7]. In the first, leaf samples were extracted in methanol, and 10 min centrifuged (10000 ×g) and the supernatant was used for measuring DPPH radical scavenging capacity. After 1 h of incubating the mixture of extract and DPPH solution (0.004%) in methanol in a dark room, the discoloration was estimated at 517 nm. The DPPH scavenging capacity of the extracts was computed by the following equation:

$$\mathrm{DPPH}\;\mathrm{radical}\;\mathrm{scavenging}\;\mathrm{activity}\left(\%\right)\;=\;\left[\left(\mathrm{Abs}\;\mathrm{control}\;-\;\mathrm{Abs}\;\mathrm{sample}\right)\rbrack/\;\left(\mathrm{Abs}\;\mathrm{control}\right)\right]\;\times\;100$$

### Statistical analysis

The experiment was executed as a split-plot experiment with a randomized complete block design (RCBD) and three replications. For ANOVA analysis, SAS software was applied, and the comparison of the means was done by Duncan’s multiple range test at *P* < 0.05. For better comprehension, a correlation heat map based on the Pearson correlation coefficient, and hierarchical cluster analysis (HCA) between treatments and variables were performed by R software (ver. 3.5.0, http://www.r-project.org).).

## Results

### Interactive effect of chitosan and water stress on biomass in two harvests

**Fig. 2:**
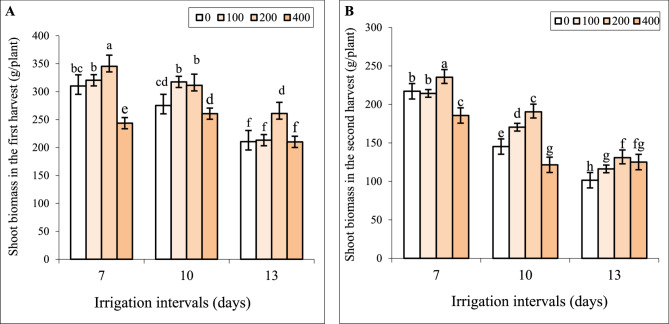
The effect of foliar-applied chitosan (0, 200, and 400 mg L^−1^) on the biomass of *Hypericum perforatum* in two harvests (**A**, **B**) under various irrigation intervals (7, 10, and 13 days). Values with the same letter have no significant difference at *P* ≤ 0.05 based on Duncan’s multiple-range tests

In the first harvest, spraying with 100 mg L^−1^ chitosan did not change, the concentration of 200 mg L^−1^ increased and 400 mg L^−1^ chitosan significantly decreased shoot dry weight (biomass) under normal (7-day interval) irrigation (Fig. 2A). With increasing irrigation intervals from 7 to 10 days (mild water stress) the reduction of biomass was insignificant, whereas shoot dry weight significantly decreased under irrigation interval of 13 days (severe water stress). Foliar application of 100 and 200 mg L^−1^ chitosan notably increased biomass under mild water stress, whereas spraying with 400 mg L^−1^ chitosan significantly decreased biomass compared to the respective control. Under severe water stress, only spraying with 200 mg L^−1^ of chitosan alleviated growth inhibition and the biomass of plants sprayed with the concentration of 400 mg L^−1^ had no significant difference from the respective control (Fig. 2A).

The negative impact of reduced irrigation was more pronounced in the second harvest than in the first harvest and both mild and severe water stress significantly decreased biomass. Foliar spraying with 200 mg L^−1^ chitosan was the best treatment and significantly increased biomass under all irrigation levels and the concentration of 400 mg L^−1^ significantly decreased biomass under 7- and 10-day irrigation intervals when compared to the respective controls notably increased biomass (Fig. 2B).

### Interactive effect of chitosan and water stress on RWC and proline accumulation

**Fig. 3:**
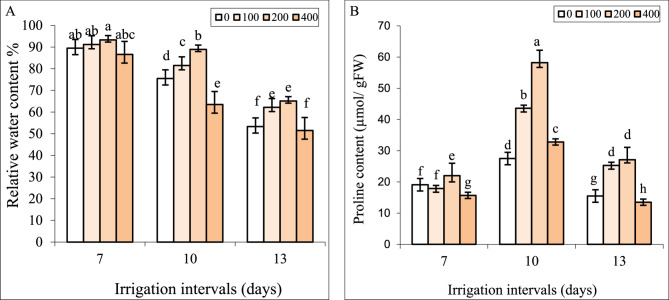
The effect of foliar-applied chitosan (0, 200, and 400 mg L^−1^) on relative water content (**A**) and proline content (**B**) in leaves of *Hypericum perforatum* under various irrigation intervals (7, 10 and 13 days). Values with the same letter have no significant difference at *P* ≤ 0.05 based on Duncan’s multiple-range tests

Under normal irrigation (7-day interval), the impact of all concentrations of chitosan on RWC was insignificant (Fig. 3). By increasing the irrigation intervals to 10 and 13 days, the concentrations of 100 and 200 mg L^−1^ of chitosan significantly increased RWC compared to respective controls in each group. In contrast, foliar spraying with 400 mg L^−1^ chitosan significantly decreased RWC under both reduced irrigation levels compared to respective controls (0 mg L^−1^ chitosan).

Under normal irrigation (7-day-interval), the effect of spraying with 100 and 200 mg L^−1^ chitosan on proline content was insignificant, whereas the concentration of 400 mg L^−1^ significantly decreased it (Fig. 3B). By increasing the irrigation intervals to 10 days whereas 13-day interval significantly reduced proline content compared to the control. Foliar spraying with 100 and 200 mg L^−1^ chitosan increased proline content under mild and severe water stress, compared to respective controls in these groups. Proline content in plants sprayed with 400 mg L^−1^ chitosan was less compared to plants treated with 200 mg L^−1^ chitosan under the irrigation intervals of 10 and 13 days (Fig. 3B).

### Interactive effect of chitosan and water stress on chlorophyll

**Fig. 4:**
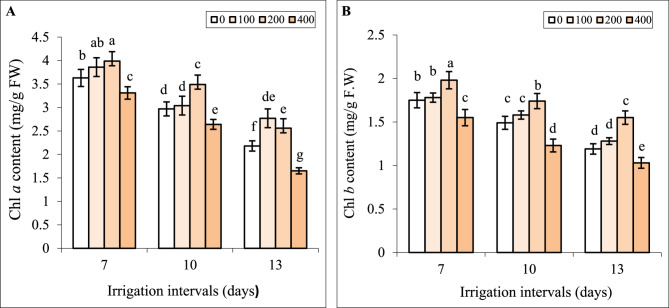
The impact of foliar-applied chitosan (0, 200, and 400 mg L^−1^) on chlorophyll *a* (**A**) and chlorophyll *b* (**B**) contents in leaves of *Hypericum perforatum* under various irrigation intervals (7, 10, and 13 days). Values with the same letter have no significant difference at *P* ≤ 0.05 based on Duncan’s multiple-range tests

Under normal irrigation (7-day interval), the effect of spraying with 100 mg L^−1^ chitosan on Chl *a*, *b* contents was insignificant, whereas the concentration of 200 mg L^−1^ significantly increased these attributes (Fig. 4A, B).

Both reduced irrigation levels significantly reduced Chl *a*, *b* contents compared to the controls. Foliar spraying with 200 mg L^−1^ chitosan increased Chl *a*, *b* contents under mild and severe water stress, compared to respective controls in these groups. Chl *a*, *b* contents in plants sprayed with 400 mg L^−1^ chitosan were less under all irrigation intervals when were compared to the respective controls (Fig. 4A, B).

### Interactive effect of chitosan and water stress on MDA and H_2_O_2_ in the leaves

**Fig. 5:**
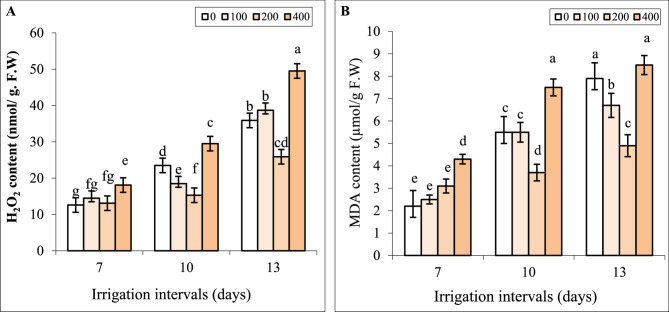
The impact of foliar-applied chitosan (0, 200, and 400 mg L^−1^) on H_2_O_2_ (**A**) and MDA (**B**) contents in leaves of *Hypericum perforatum* under various irrigation intervals (7, 10, and 13 days). Values with the same letter have no significant difference at *P* ≤ 0.05 based on Duncan’s multiple-range tests

Under normal irrigation (7-day interval), spraying with 100 and 200 mg L^−1^ chitosan did not change the content of H_2_O_2_ and MDA, while 400 mg L^−1^ chitosan significantly increased these attributes compared to control (Fig. 5A, B).

The content of MDA and H_2_O_2_ significantly augmented in leaves under both reduced irrigation levels. Foliar spraying by 200 mg L^−1^ chitosan significantly decreased MDA and H_2_O_2_ contents in both reduced irrigation levels. In contrast, foliar application of 400 mg L^−1^ chitosan significantly increased the content of MDA and H_2_O_2_ in the leaves of plants exposed to mild water stress. Under severe water stress, in leaves sprayed with 400 mg L^−1^ chitosan H_2_O_2_ concentration was significantly higher and MDA content was equal to the respective control (Fig. 5A, B).

### Interactive effect of chitosan and water stress on antioxidant enzyme activity

**Fig. 6:**
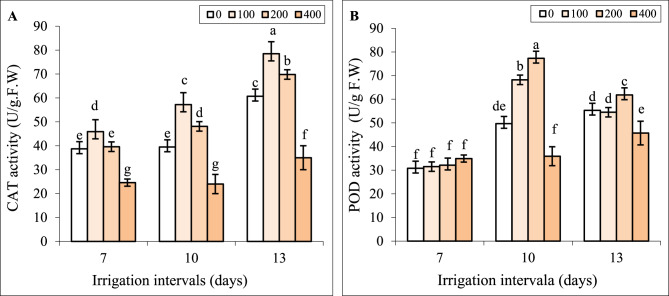
The impact of foliar-applied chitosan (0, 200, and 400 mg L^−1^) on CAT (**A**) and POD (**B**) activity in leaves of *Hypericum perforatum* under various irrigation intervals (7, 10, and 13 days). Values with the same letter have no significant difference at *P* ≤ 0.05 based on Duncan’s multiple-range tests

Under normal irrigation (7-day interval), spraying with 100 and 200 mg L^−1^ chitosan did not change the activities of POD and CAT, while 400 mg L^−1^ chitosan significantly decreased CAT activity compared to control (Fig. 6A, B).

The CAT activity did not change but POD activity significantly increased in leaves under mild water stress. However, the activities of both antioxidant enzymes were significantly enhanced under severe water stress. Foliar spraying with chitosan, especially at the concentration of 200 mg L^−1^ significantly increased the activities of POD and CAT enzymes, and contrary, the concentration of 400 mg L^−1^ decreased the activities of these enzymes under both reduced irrigation levels (Fig. 6A, B).

### Interactive effect of chitosan and water stress on hypericin concentration

**Fig. 7:**
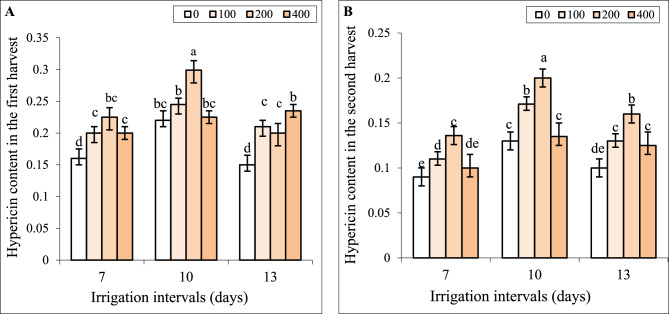
The impact of foliar-applied chitosan (0, 200, and 400 mg L^−1^) on hypericin content in leaves of *Hypericum perforatum* under various irrigation intervals (7, 10, and 13 days). Values with the same letter have no significant difference at *P* ≤ 0.05 based on Duncan’s multiple-range tests

Our results showed that by increasing the irrigation intervals to 10 days the content of hypericin increased in the first and second harvests. However, by increasing the irrigation interval to 13 days, hypericin content significantly decreased and there is no significant difference relative to the control in both harvests (Fig. 7A, B). Foliar spraying with the concentrations of 100, and 200 mg L^−1^ chitosan increased hypericin content compared to respective controls in each group in both harvests. Under all irrigation levels, hypericin contents in plants treated with 400 mg L^−1^ chitosan was less compared to plants sprayed with 200 mg L^−1^ chitosan in both harvests. In many cases, hypericin contents in these plants were equal to respective controls in each group, except in plants treated with 400 mg L^−1^ chitosan and a 13-day irrigation interval (Fig. 7A, B).

### Interactive effect of chitosan and water stress on TPC and DPPH scavenging capacity

**Fig. 8:**
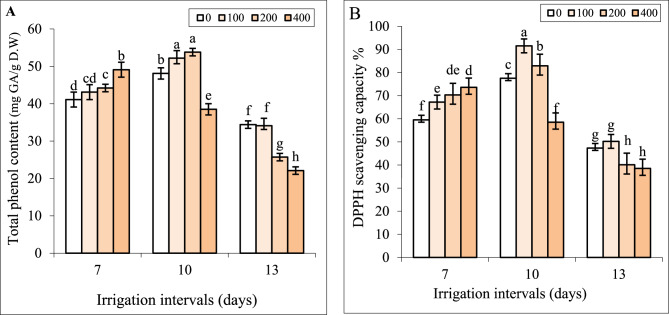
The impact of foliar-applied chitosan (0, 200, and 400 mg L^−1^) on TPC (**A**) and DPPH scavenging capacity (**B)** in leaves of *Hypericum perforatum* under various irrigation intervals (7, 10, and 13 days). Values with the same letter have no significant difference at *P* ≤ 0.05 based on Duncan’s multiple-range tests

Under normal irrigation (7-day interval), spraying with 100 mg L^−1^ chitosan did not change TPC, and significant increments of TPC were recorded in plants sprayed with 200 and 400 mg L^−1^ chitosan (Fig. 8A).

Our results showed that by increasing the irrigation intervals to 10 days TPC increased while in the 13-day interval, this attribute significantly decreased and was less than the control. Foliar spray with 100 and 200 mg L^−1^ chitosan significantly increased TPC under mild water stress. As shown in Fig. 7, TPC did not change by foliar application of 100 mg L^−1^ chitosan under severe water stress. The values of TPC in plants treated with 400 mg L^−1^ chitosan were lower than those in plants sprayed with 200 mg L^−1^ chitosan under both reduced irrigation levels and were less than respective controls in each group (Fig. 8A).

Under the irrigation intervals of 7 and 10 days, significant increments of DPPH scavenging capacity were recorded in plants sprayed with 100 and 200 mg L^−1^ chitosan (Fig. 8B). When the irrigation interval increased to 13 days, spraying with 100 and 200 mg L^−1^ chitosan DPPH scavenging capacity was less or equal to the respective control. The values of DPPH scavenging capacity in plants treated with 400 mg L^−1^ chitosan were less than respective controls under both reduced irrigation levels whereas these values were higher than control under normal (7-day-interval) irrigation (Fig. 8B).

### Multivariate analysis

**Fig. 9:**
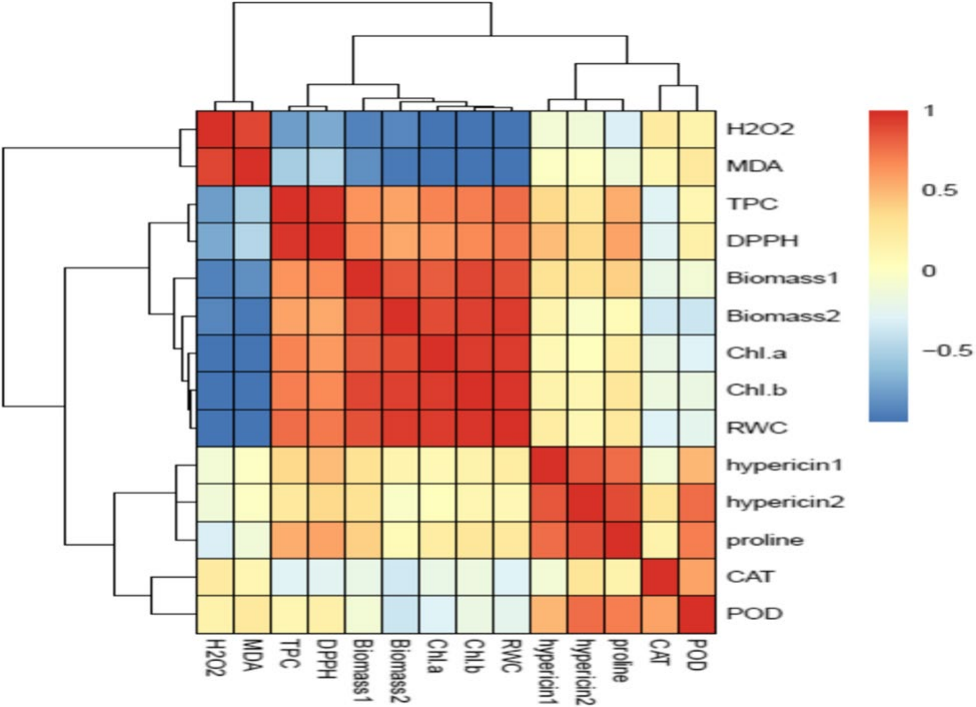
Heat map based on Pearson’s coefficient correlations between all variables in *Hypericum perforatum. *Strong positive and negative correlations are represented by dark red and dark blue colors, respectively. The investigated variables include: biomass in harvest 1, 2 (biomass1, 2), hypericin in harvest 1, 2 (hypericin 1, 2), chlorophyll a, b contents (Chl a, Chl b), malondialdehyde content (MDA), hydrogen peroxide concentration (H_2_O_2_), Proline concentration (Proline), Catalase activity (CAT), peroxidase activity (POD), total phenol content (TPC), DPPH scavenging capacity (DPPH).

The heat map obtained based on Pearson’s correlation between traits (Fig. 9) indicated that RWC was positively correlated with biomass in harvest 1, 2, Chl *a*, and *b* contents, TPC, and DPPH scavenging capacity in plants. There was a strong and negative correlation between RWC with MDA and H_2_O_2_ contents. The MDA and H_2_O_2_ contents negatively correlated with biomass 1, 2, Chl contents, TPC, and DPPH scavenging capacity (Fig. 9).

**Fig. 10:**
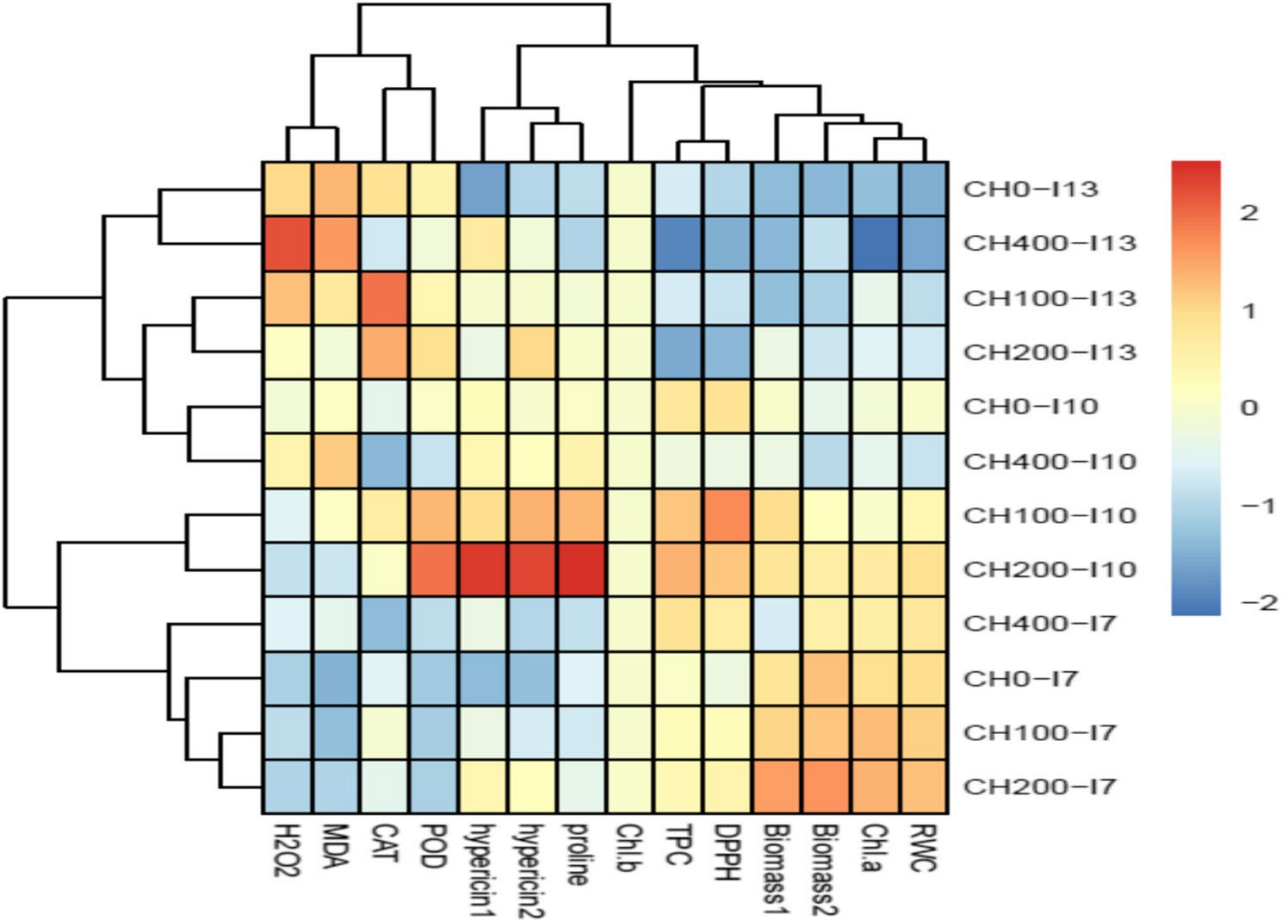
Visualization of the interactions between treatments and variables via a hierarchically clustered heat map. Please see the abbreviation of variables in the capture of Fig. 8. The treatments were included: control (CH0-I7), 100 mg L^−1^ chitosan + 7-day irrigation interval (CH100- I7), 200 mg L^−1^ chitosan + 7-day irrigation interval (CH200- I7), 400 mg L^−1^ chitosan + 7-day irrigation interval (CH400- I7), without chitosan + 10-day irrigation interval (CH0- I10), 100 mg L^−1^ chitosan + 10-day irrigation interval (CH100- I10), 200 mg L^−1^ chitosan + 10-day irrigation interval (CH200- I10), 400 mg L^−1^ chitosan + 10-day irrigation interval (CH400- I10), without chitosan + 13-day irrigation interval (CH0- I13), 100 mg L^−1^ chitosan + 13-day irrigation interval (CH100- I13), 200 mg L^−1^ chitosan + 13-day irrigation interval (CH200- I13), 400 mg L^−1^ chitosan + 13-day irrigation interval (CH400- I13).

The results of hierarchical clustering analysis (HCA in Fig. 10) showed that the concentration of 100 and especially 200 mg L^−1^ chitosan increased biomass in harvest 1, 2, Chl *a* content, and RWC under normal irrigation (CH100-I7, CH200-I7). While, these chitosan concentrations further affected the activities of antioxidant enzymes, H_2_O_2_ and MDA contents, and hypericin 1,2 in leaves under both reduced irrigation levels (CH100-I10, CH200-I10, CH100-I13, CH200-I13). In contrast, treatments of CH400-I10, CH400-I13 respectively were clustered with CH0-I10 and CH0-I13, and increased MDA contents and negatively influenced biomass in harvest 1, 2, Chl *a* content, RWC, TPC, and DPPH scavenging capacity.

## Discussion

Water deficit stress is one of the most common environmental concerns worldwide that can decrease the productivity and quality of medicinal plants [2]. In the present study, the reduced irrigation decreased shoot biomass of St John’s Wort in both harvests (Fig. 2) which agreed with findings on *Thymus daenensis* [49] and *Foeniculum vulgare* Mill [4]. The reduced water uptake from the soil by plants results in decreased turgor and viscosity in cells which in turn diminishes cell division and expansion and consequently reduces vegetative growth parameters and fresh weight [2–4]. Furthermore, water deficit motivates abscisic acid (ABA) generation in plants which stimulates stomata closure to reduce water transpiration. At the same time, the inflow of carbon dioxide into leaves via stomata decreases and leads to less production of dry biomass due to photosynthesis’s restricted capability [1, 3]. During the second growing season, plants were subjected to elevated temperatures and reduced humidity and precipitation levels, as outlined in Table 1. These environmental conditions exacerbated the negative impact of water deficit on growth, resulting in significantly lower biomass production during the second harvest compared to the first (Fig. 2A, B). This observation underscores the necessity of irrigation with fewer intervals or more efficient strategies to mitigate the adverse effects of such environmental factors during the second season. However, foliar spraying with chitosan had favorable effects on the biomass of plants in both harvests and the best growth recovery was recorded by the concentration of 200 mg L^−1^ chitosan under all irrigation intervals (Fig. 2A, B). This indicates that this biopolymer is capable of regulating plant growth and conferring water deficit tolerance in St John’s Wort. The stimulatory effect of chitosan on upregulating the expression of genes related to photosynthesis, protein metabolism, and phytohormones levels (such as gibberellin or auxin) can improve growth parameters [30, 50, 51]. Previous studies also revealed that chitosan improved growth in chrysanthemum [52], and *Dracocephalum kotschyi* [41], by enhancing water and essential nutrient uptake in these plants.

Considering the positive correlation between RWC with biomass (Fig. 9) and HCA results (Fig. 10), it seems that higher levels of RWC in plants sprayed with 100 and 200 mg L^−1^ chitosan (Fig. 3A) retained safeguarding turgor and played a crucial role in improving the biomass, particularly under non-stress conditions and mild water stress. Chitosan as an antitranspirant improves water retention, adjusts stomatal conductance and transpiration rate, and at the same time enhances water absorption by increasing root growth which results in improved water status in plants [21]. The beneficial effect of chitosan on root growth and RWC in *Cichorium intybus* L [40]. and adjusting stomatal conductance and transpiration rate in pot marigolds [25] have been documented earlier. HCA analysis (Fig. 9) showed that 200 mg L^−1^ chitosan ameliorated osmotic stress induced by reduced irrigation was closely linked to the accretion of proline accumulation. Similar to our findings (Fig. 3), the foliar application of chitosan enhanced proline contents, resulted in improving relative water content and water-use efficiency, and consequently increased the total yield of lettuce under limited irrigation [53]. Proline accumulation is often known as the primary response of many plants to drought stress [54]. Proline plays a crucial role in osmo-protection and reduces the water potential of tissues, especially in leaves, thereby enabling plants to prevent water loss and/or continue acquiring water from soil under water deficit conditions [55]. Likely, chitosan stimulated the biosynthesis of proline to achieve osmotic adjustment and maintain turgor pressure and leaf water content in St John’s Wort under reduced irrigation. Elansary et al. [52] reported that foliar spray with chitosan, particularly in combination with robinin, improved leaf water content in water-stressed *Chrysanthemum morifolium* by higher accumulation of K^+^, Ca^+2^, carbohydrates, and proline, and upregulation of genes involved in proline biosynthesis such as pyrroline-5-carboxylate synthetases (*P5CS*), and pyrroline-5-carboxylate reductase (*P5CR*).

The positive association of Chl contents and biomass along with the negative correlation between Chl contents with H_2_O_2_ and MDA contents (Fig. 9) suggests that oxidative damage of Chl under water deficit might be responsible for declining photosynthesis and consequently biomass. Water stress can decrease the activity of enzymes involved in chlorophyll biosynthesis, or may lead to the destruction of light-harvesting protein complexes and ROS-mediated degradation of chlorophyll [2, 3]. The positive effect of chitosan on Chl content (Fig. 4A, B) might be related to either increasing the internal cytokinin level and accordingly the chlorophyll synthesis or suppressing ethylene and chlorophyllase activity and reducing chlorophyll destruction in leaves [56]. In the present study, foliar spray with chitosan reduced ROS generation (as verified by reducing H_2_O_2_ in leaves in Fig. 5A) under reduced irrigation which in turn contributed to preserving chlorophyll in leaves of St John’s Wort. This alleviation of oxidative damage of Chl. likely enhanced photosynthesis and increased biomass under both levels of reduced irrigation. Likewise, foliage-applied chitosan increased chlorophyll content, photosynthesis, and growth parameters of *Origanum majorana* [39] and *Cichorium intybus* L [40]. under water deficit stress.

The disruption of metabolic processes in tissues under reduced irrigation appraised ROS generation and, as a consequence increased membrane lipid peroxidation. Foliar application of chitosan significantly reduced H_2_O_2_ and MDA contents (Fig. 5A, B). It is known that chitosan due to having a large number of hydroxyl and amino groups in its structure can directly react with OH and O_2_^_^ radicals and convert ROS to nontoxic forms [20]. Based on HCA (Fig. 10) and our data, the positive effect of 200 mg L^−1^ chitosan on reducing oxidative stress was correlated with enhancing the activities of POD and CAT enzymes and proline content. Similar to our findings (Fig. 3B), chitosan-induced proline accumulation has been reported in *Thymus daenensis* [24] and *Hyssopus officinalis* L [24]. under reduced irrigation. Proline not only can act as an osmoprotectant but also is involved in quenching of ROS and maintenance of redox balance under stressful conditions [55]. The increased activity of antioxidant enzymes (Fig. 6) also enhances ROS scavenging and reduces lipid peroxidation (Fig. 5A, B). The stimulatory effect of chitosan on proline accumulation and the activity of antioxidant enzymes might be related to the induction of signals and high transcription of respective genes [23, 52]. Al-Ghamdi [39] reported that chitosan treatment increased expression of *MnSOD Cu*/*ZnSOD*,* FeSOD*,* APX*,* DREB2* and *ERF3* s and higher activity of SOD and APX enzymes reduced ROS accumulation and oxidative stress in *Origanum majorana* under water stress.

The increasing evidence has shown that chitosan can act as a potent elicitor and stimulate secondary metabolism in plants [15]. Herein, the foliage applied chitosan promoted the biosynthesis of TPC, and hypericin (Figs. 7 and 8), which corresponded to DPPH scavenging capacity in leaves (Fig. 8) under a normal irrigation regime. This indicated that chitosan improved the pharmaceutical value and antioxidant activity of St John’s Wort. Likely, enhanced Chl content and photosynthetic efficiency induced by chitosan, increased access to carbon precursors for producing hypericin and phenolic compounds. Kahromi and Khara [41] also reported that foliar-applied chitosan increased TPC, and the content of apigenin, rosmarinic acid, and quercetin in the medicinal plant *Dracocephalum kotschyi*.

The phenolics are antioxidant compounds that can contribute to ROS scavenging under various stresses [4–8]. In this study also the positive correlation between TPC and DPPH (Fig. 8) and the negative correlation between these attributes with H_2_O_2_ and MDA content affirmed this opinion. Although, at moderate water stress the concentration of phenolics increased to enhance antioxidant defense ability (Fig. 8A, B), severe water stress decreased TPC and was accompanied by an increased level of oxidative stress (Fig. 5) in this herb. Gray et al. [38] also reported that when St John’s Wort plants were exposed to brief drought stress during both flowering and seed development, the concentration of rutin, quercetin, pseudohypericin, hypericin, quercitrin, and chlorogenic acid increased, contrary severe drought stress decreased the contents of some phytochemicals. Likely, closing stomata and limited CO_2_ assimilation reduced carbon allocation toward secondary metabolism under severe water stress [2, 4]. In addition, high oxidative stress induced by severe water stress (Fig. 5) might harm the enzymes involved in the biosynthesis of hypericin and phenolic compounds, resulting in decreased levels of these metabolites and impaired DPPH scavenging capacity in leaves. Similar trends were observed in *Salvia officinalis*, where moderate salt stress increased TPC, TFC, rosmarinic acid content, and DPPH scavenging activity, whereas high salinity caused significant declines in these attributes [7]. Foliar application of chitosan increased the content of hypericin and phenolic compounds under both reduced irrigation levels (Figs. 7 and 8A) which coincided with higher DPPH scavenging capacity (Fig. 8B) as well as lower H_2_O_2_ and MDA content in leaves of these plants under water stress (Fig. 5). Likewise, the chitosan treatment (0.2 and in particular 0.4 g L^−1^) improved plant growth parameters and enhanced total phenol content and DPPH scavenging activity in two species of *Ocimum basilicum* and *Ocimum ciliatum* under water deficit stress [57]. It is known that chitosan can increase phenolic content due to motivating the activity of enzymes involved in the phenylpropanoid pathway such as phenylalanine ammonia-lyase (PAL) and tyrosine ammonia-lyase [19, 58, 59].

In the current study, the effects of chitosan on all attributes of St John’s Wort were dependent on its concentration. In many cases, applying 200 mg L⁻¹ chitosan resulted in the most favorable effects in terms of secondary metabolism and growth recovery under water stress (Figs. 1, 2, 3, 4, 5, 6, 7 and 8). However, a higher concentration of 400 mg L⁻¹ significantly reduced biomass (Fig. 2) and chlorophyll content (Fig. 4), while increasing H₂O₂ content and lipid peroxidation (Fig. 5), even under non-stress conditions. All these effects are recognized as the symptoms of a stressful situation for the plants. Another study showed that a high chitosan dose inhibited growth and biomass accumulation but increased the levels of valine, isoleucine, glutamine, γ-aminobutyric acid, fructose, sucrose, polyunsaturated fatty acids, epicatechin, xanthones, dimethylallyl pyrophosphate, and stigmasterol in *H. perforatum* in vitro roots [60]. A review of the scientific literature revealed an extensive variation in optimal or harmful chitosan concentrations across plant species. For instance, foliar application of chitosan at 2.5 and 5.0 g L⁻¹ enhanced growth parameters and antioxidant activity in *Hyssopus officinalis* L. plants under varying irrigation intervals [24]. In contrast, treatment with 50 mg L⁻¹ chitosan inhibited growth, saponin content, and steroid accumulation, particularly in roots of *Calendula officinalis* plants grown in pots and also in hairy root cultures of the same species [61]. Similar inhibitory effects of chitosan on biomass have been reported for Dendrobium [62], cordyline [63], and pot marigold [25] at concentrations of 1250, 100, and 10 mg L⁻¹ respectively. This extensive variability in outcomes across studies likely stems from differences in several factors: the type of chitosan (molecular weight and degree of de-acetylation), its concentration and application method (soil, foliar), plant species, developmental stage, and experimental conditions. Lopez-Moya et al. reported that lower doses of chitosan (0.01–0.1 mg mL⁻¹) had minimal impact on Arabidopsis growth, while higher concentrations (> 0.5 mg mL⁻¹) severely inhibited its root development. This inhibition was linked to modification of the transcription factor WOX5, auxin overproduction in roots, and the overexpression of genes related to jasmonic acid and salicylic acid biosynthesis and signaling pathways [64]. Therefore, the contrast effects of chitosan at 200 mg L⁻¹ and 400 mg L⁻¹ concentrations on St John’s Wort might also be attributed to alterations in stress-related gene expression and hormonal regulations. Future research should validate the accuracy of this hypothesis. Another possible explanation can be the excessive ROS generation induced by higher concentrations of chitosan. Herein, applying 400 mg L^−1^ chitosan increased H_2_O_2_ content and lipid peroxidation in leaves under all irrigation intervals (Fig. 5). The close association of the highest H_2_O_2_ and MDA contents with treatments of CH400-I10, CH400-I13 in HCA (Fig. 10) also further supported above conclusion. Previous studies also revealed that chitosan treatment induced H_2_O_2_ generation in sweet peppers and Arabidopsis [65, 66]. Cabrera et al. [67] and Singh [68] reported that high chitosan concentrations can impair growth in spinach plants and Arabidopsis cell suspensions due to excessive ROS production and oxidative stress imbalance. It is known that the lower levels of ROS especially H_2_O_2_ can act as a signal molecule to promote protective responses in plants, whereas high concentrations of ROS lead to oxidative damage under various stresses [7, 43, 69]. In the current study also the beneficial effects of optimal concentration of chitosan (e.g., 200 mg L⁻¹) might be linked to a fine-tuned ROS signaling mechanism that promotes growth and triggers the biosynthesis of hypericin, phenolic compounds, and antioxidant enzymes. In contrast, higher concentrations of chitosan result in excessive ROS accumulation, disrupting these responses and leading to oxidative damage and the decrease of biomass.

## Conclusion

This research highlights the beneficial role of chitosan in enhancing water stress tolerance and promoting secondary metabolism in St John’s Wort. Foliar application of chitosan at concentrations of 100 and 200 mg L⁻¹ effectively reduced oxidative stress caused by water deficit by enhancing antioxidant enzyme activity and increasing the accumulation of phenolic compounds. Notably, the 200 mg L⁻¹ concentration exhibited the most pronounced effect on total phenolic content, hypericin levels, and overall antioxidant capacity under both normal and water-limited conditions. However, higher concentrations, such as 400 mg L⁻¹, were found to diminish these beneficial effects, underscoring the necessity of optimizing chitosan dosage for maximum efficacy. Future research should explore the molecular mechanisms underlying these effects, including the role of stress-related gene expression and phytohormone modulation, to better understand the dual role of chitosan in plant stress physiology. This finding emphasizes the importance of species-specific studies to determine the appropriate concentration, application method (e.g., foliar or soil), frequency, and timing relative to plant developmental stages. While further investigation is required to establish precise guidelines, this study reinforces the potential of chitosan as a valuable biostimulant to enhance the yield and quality of medicinal plants under water-stress conditions.

## Data Availability

The data that support the findings of this study are available from the second author upon reasonable request.
